# Letter to the editor regarding the article: “All-inside meniscal repair surgery: factors affecting the outcome” by Haroon Majeed et al.

**DOI:** 10.1007/s10195-017-0454-y

**Published:** 2017-03-13

**Authors:** Alexander Zimmerer

**Affiliations:** ARCUS Sportklinik Pforzheim, Rastatterstr. 17-19, 75179 Pforzheim, Germany

To the Editor,

I read the study “All-inside meniscal repair surgery: factors affecting the outcome” by Majeed et al. [1] with great interest.

The authors assessed the outcome of meniscal repair surgery and the role of anterior cruciate ligament reconstruction. They found that meniscal repair failure rate was higher in the delayed anterior cruciate ligament (ACL) reconstruction group compared to patients who had ACL reconstruction performed early. One of their conclusions was “Our results have shown that the outcome of meniscal repair is statistically significantly better if ACL reconstruction is performed simultaneously with the meniscal repair (*p* = 0.0006)”.

However, I noted irregularities in these results. According to the data, ligament injuries were found in 83 patients along with meniscal tears. “Of these 83 patients with ruptured ligaments, 55 had simultaneous ACL reconstruction or reconstruction performed within six weeks of injury, while 26 had their ACL reconstructed at a later stage following an initial meniscal repair (after six weeks of injury)”. This results in 81 patients having had ACL reconstruction.

The authors found “that patients who had ACL reconstruction performed early (…) had a meniscal repair failure rate of 14.5% (*n* = 12)”. However, if the 12 patients referred to the number of patients with early ACL reconstruction (*n* = 55), the proportion is 21.8% (12/55). Similarly, the proportion of patients with delayed ACL reconstruction does not seem to be correct. “In comparison, patients who had delayed ACL reconstruction (…) had a meniscal repair failure rate of 27% (*n* = 22; *p* = 0.0006)”. Referring the 22 patients to the total number of patients with delayed ACL reconstruction (*n* = 26), the proportion should be 84.6% (22/26). The percentages given in the article refer to the total number of patients who had ACL reconstruction (*n* = 81, 12/81 = 14.5%; 22/81 = 27%).

In Table 1 [1] (Summary of outcomes for patients who underwent meniscal repair) the success percentage is given as 86% in the simultaneous ACL reconstruction group and 77% in the delayed ACL reconstruction group. This would result in 47 patients with successful meniscal repair and 8 patients with failed repair in the simultaneous ACL reconstruction group, and 20 patients with successful meniscal repair and 6 patients with failed repair in the delayed ACL reconstruction group.

By taking these numbers into consideration, there still might be a statistically significant difference; however, the numbers should certainly be recalculated to state the given conclusion in the article.

**Note from the Editorial Office:** the Corresponding Author of the article commented on (H.M.) was invited to submit a response letter, which would have been published. The Author preferred to contact the Executive Editor by mail to confirm the miscalculation, that nevertheless would not have influenced the conclusions.
